# Dr. Richardson, F.R.S., as a Novelist

**Published:** 1889-07-13

**Authors:** 


### DR. RICHARDSON, F.R.S., AS A NOVELIST.

When an author of Dr. Richardson's fame writes a story* for our instruction or for our amusement, we have a right to expect that the work shall be in every particular far removed from the commonplace, and in "The Son of a Star" we are not disappointed in our expectations. It is not so much a novel, in the ordinary acceptation of the term, as a series of living pictures, which the author tells us are dreams, and in this dreamland we get an approach to one at least of the old classic unities. As the panorama is unrolled, we see passing before us the outline of a grand period in the world's history. It brings, too, vividly to our memories that wonderful epoch—the Roman occupation of Britain.

The reign of the Emperor Hadrian is remarkable as the time when the Roman power culminated, and it is also remarkable for the suppression of a revolt of the Jews, and for the final dispersion of that extraordinary race. Hadrian wished to rebuild Jerusalem. This excited the jealousy of the Jews, who rose in insurrection, and who received a terrible punishment at the hands of the Romans. It is said that over half a million were slain.

This is the period in which Dr. Richardson has cast his story, and his chief characters are the leading actors in that awful drama; but, excepting Hadrian and Akiba, they are all his own creations, or, at most, bear the old names. In our first dream we see the Roman camp in Britain prepared for some unusual exhibition, the onlookers being chiefly Romans and Britons with apparently a sprinkling of Jews, and two individuals—a father and daughter—from the Island of Juverna. Then we notice the sudden arrival of the peripatetic emperor, the passage round the arena of the Centurion Fidelis, who has lived a hundred years, the combat between a Numidian slave and wolves; and, lastly, Simeon, the living torch, bursting through the camp and flying o'er the hills until he looks like a mere speck of fire in the distance. The saving of Simeon's life by Erine and her father, and the speedy falling in love of the young couple, are very finely detailed. A halo of romance is thrown round the Island of Juverna. It was at that time an isle of peace and beauty—an earthly Paradise from which evil had been expelled. The inhabitants ate no animal food, and drank nothing but water. They have fairly well adhered to the abstinence from meat, but the wine of the country has taken the place of the crystal fount not with advantage to the race. And the beauty of the island has been sadly marred. The round towers, on which Erine's people are said to have lighted their sacred fires, still stand; but the forests that once surrounded them are shorn to the ground.

The scene portraying the death of the aged centurion is very touching. Saserna meets Typhon the Asclepiad at the tent of Fidelis, and asks how the old centurion is:

"Dost thou remember near to Rome a lofty pedestal at foot of the Aventine Hills?"

"I remember it well; it was raised by our great fathers of the State to the goddess of health, Salus, and it stood there for ages defying time."

"It did, but now knowest thou its fate?"

"Not more than I have told you, illustrious healer."

"Then I will tell you more. One morning, a month or so after we left Rome for this remote island, that statue stood in all its apparent strength and in all its beauty. It was the day of the festival dedicated to the goddess, and in honour of the event a smart and handsome youth was chosen to climb the statue and place a garland on her brow. A hundred times before the same ceremony had been performed; again the rite was fully carried out, and again at night the sun cast his parting rays on the garlanded Salus. But in the night there arose a storm, and when the sun threw his glory once more on the charmed spot, the statue of Salus lay as dust at the foot of its crumbling pedestal, the garland buried in the ruins."

"Alas! Alas! And is that the fate of Fidelis.'

Just as every parent has his pet child, so every author has his pet volume, and probably a pet chapter in the volume. In the sixteenth chapter of "The Son of the Star" we feel the author's presence as we read. We dream on with all our senses entranced, and awake with regret when the dream is over. There is a wonderful description of a wonderful group, and the art-beauty of the scene ending the chapter is very fine. It reminds us of the Easter-morn scene in the Gothic library of "Faust," and it is no light thing to say that it will bear comparison with that scene. Some of the minor incidents are very pleasing, and they linger in our memory. For instance, the restoration of the power of Segonax the potter; the description of the library of Akiba, his arrival in Jerusalem and the contrast between his anticipation of its grandeur and the reality, and the meeting between Akiba and the Princess Tyra.

We must pass over the fate of Akiba. We cannot quote more of the volume, nor can we follow Simeon through his brief career of glory, nor even through his ingenious escape from the Romans and his union with Erine.

There is much mental food in "The Son of a Star," and the fact that a new edition has been so soon called for shows that Dr. Richardson's venture into imaginative literature has been appreciated.

* "The Son of a Star." By W. B. Richardson. Longmans and Co. One volume, pp. 470.

